# Severe endometriosis during pregnancy: incarcerated gravid uterus with concurrent placenta previa

**DOI:** 10.1016/j.xagr.2025.100463

**Published:** 2025-02-21

**Authors:** Mariya Kobayashi, Kosuke Hiramatsu, Tateki Tsutsui

**Affiliations:** a Department of Obstetrics and Gynecology, Graduate School of Medicine, The University of Osaka, Suita, Osaka, Japan (Kobayashi and Hiramatsu); b Department of Obstetrics and Gynecology, Japan Community Healthcare Organization Osaka Hospital, Osaka, Japan (Kobayashi and Tsutsui)

## Case presentation

**Figure 1:**
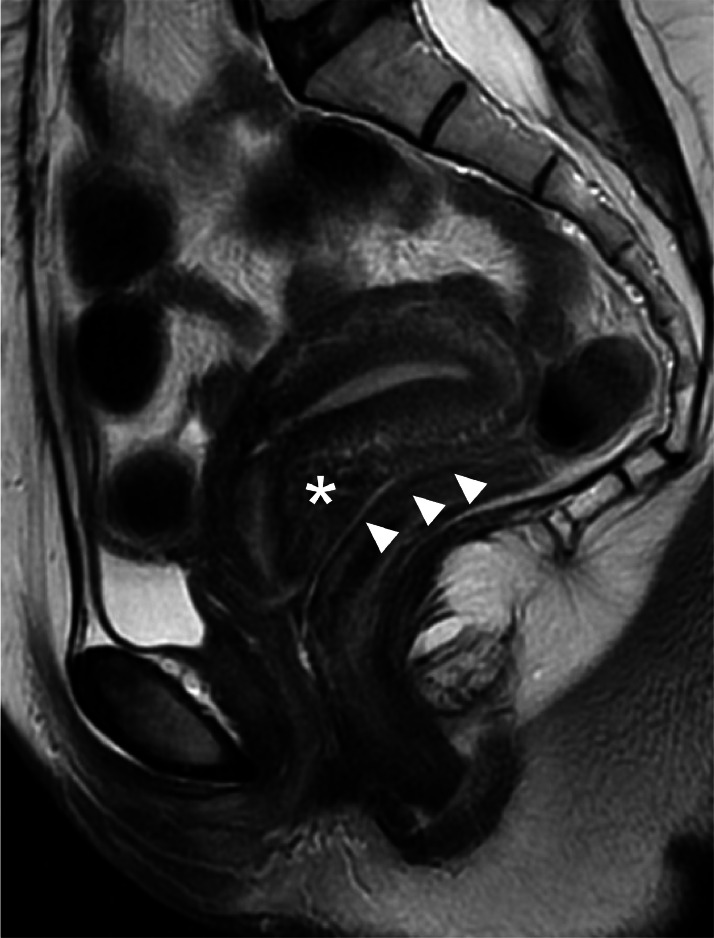
Prepregnancy magnetic resonance imaging Sagittal T2-weighted image showing a retroflexed uterus. The elevated posterior vaginal fornix (*) and the absence of free peritoneal fluid in the Douglas pouch (*arrowhead*) suggest posterior cul-de-sac obliteration associated with severe endometriosis.

**Figure 2:**
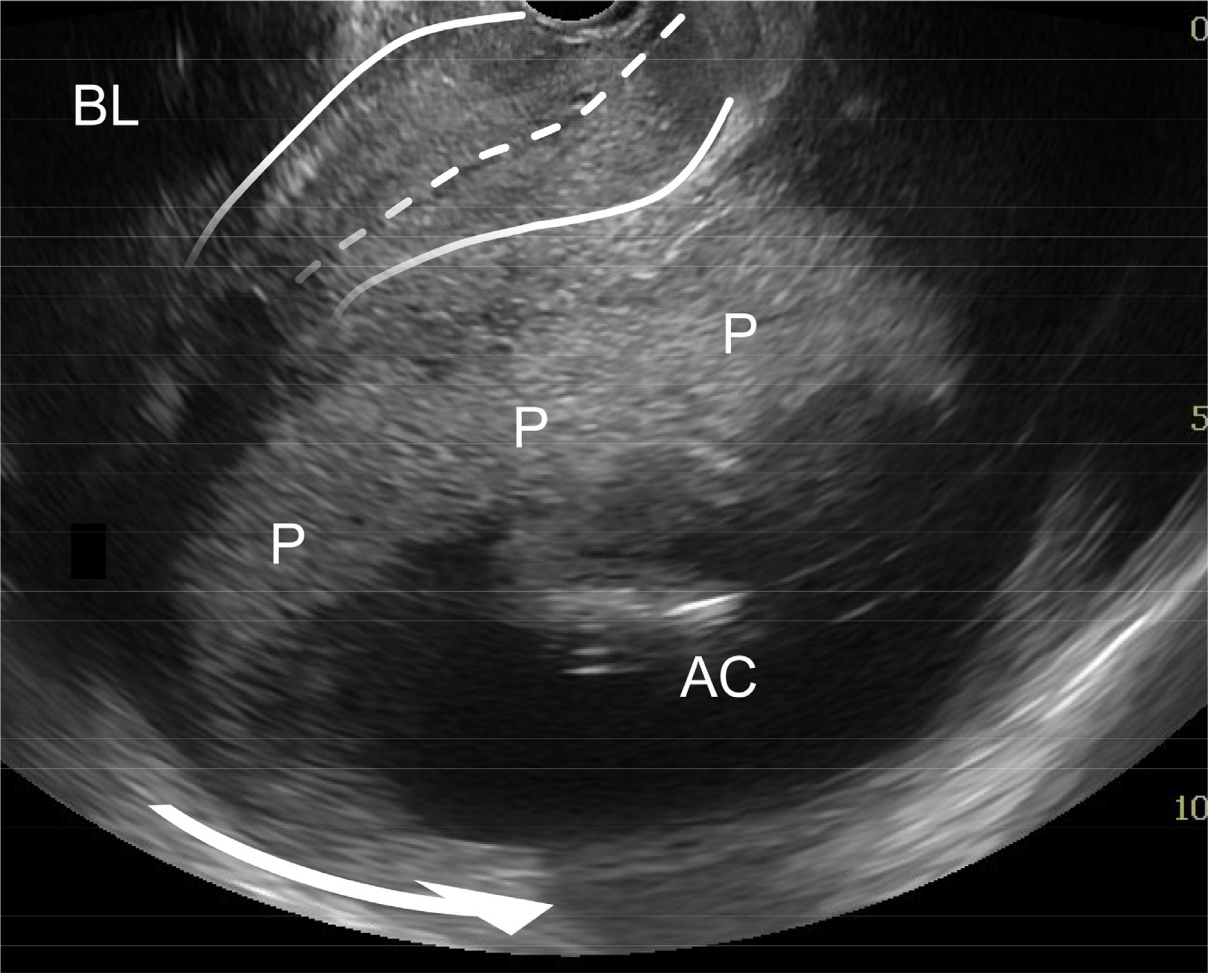
Transvaginal ultrasound at 16 weeks of gestation Transvaginal ultrasonography showing an elongated cervix (*solid* and *dotted lines*) and significant cranial displacement of the internal cervical os above the symphysis pubis caused by a retroflexed incarcerated gravid uterus (*arrow*). The placenta is attached to the anterior wall. However, determining the positional relationship between the internal cervical os and the placenta is difficult via transvaginal ultrasonography because of the elongated cervix, which complicates the diagnosis of placenta previa. *AC*, amniotic cavity; *BL*, bladder; *P*, placenta.

**Figure 3:**
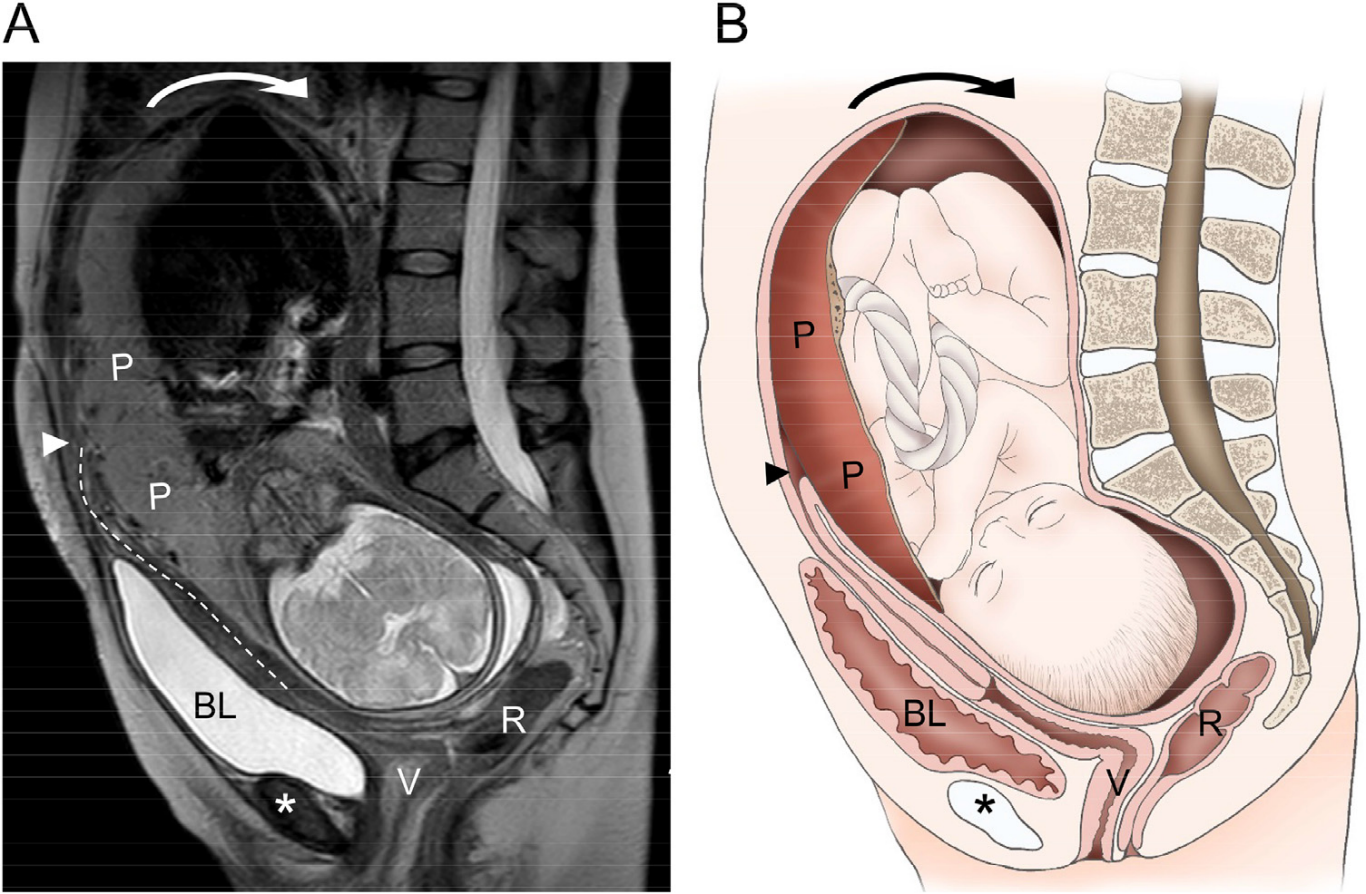
Magnetic resonance imaging at 33 weeks of gestation **A,** Sagittal T2-weighted image showing the coexistence of a retroflexed IGU and placenta previa. The uterus is heavily retroflexed (*arrow*), and the cervix seems elongated (*dotted line*), resulting in the displacement of the internal cervical os (*arrowhead*) above the symphysis pubis (*). The placenta obscured the internal cervical os. **B,** The image shows a schematic representation of the IGU complicated by placenta previa. *BL*, bladder; *IGU*, incarcerated gravid uterus; *P*, placenta; *R*, rectum; *V*, vagina.

A 38-year-old female patient with a history of cesarean delivery (CD) and severe endometriosis (Figure 1) conceived spontaneously. At 16 weeks of gestation, transvaginal ultrasonography revealed a retroflexed incarcerated gravid uterus (IGU) (Figure 2). Repositioning was avoided because of the risk of hemoperitoneum from severe adhesions in the pouch of Douglas caused by endometriosis potentially associated with the IGU. Magnetic resonance imaging at 33 weeks of gestation revealed an IGU with complete placenta previa (Figure 3). A planned CD was performed at 35 weeks of gestation because of the possibility of a placenta accreta spectrum resulting from the placenta overlying the previous CD scar. Intraoperative ultrasonography facilitated the navigation of complex anatomic relationships, and the newborn was delivered via a vertical uterine incision with spontaneous placental delivery.

Kobayashi. Incarcerated gravid uterus with concurrent placenta previa. Am J Obstet Gynecol Glob Rep 2025.

Kobayashi. Incarcerated gravid uterus with concurrent placenta previa. Am J Obstet Gynecol Glob Rep 2025.

Kobayashi. Incarcerated gravid uterus with concurrent placenta previa. Am J Obstet Gynecol Glob Rep 2025.

Endometriosis, along with previous CDs, is recognized as a risk factor for placenta previa.^1^^,^^2^ In this case, severe endometriosis may have contributed to the coexistence of IGU and placenta previa.

## CRediT authorship contribution statement

**Mariya Kobayashi:** Writing – review & editing, Writing – original draft, Visualization, Resources, Methodology, Investigation, Data curation. **Kosuke Hiramatsu:** Writing – review & editing, Resources, Project administration, Methodology, Conceptualization. **Tateki Tsutsui:** Writing – review & editing, Supervision, Resources, Conceptualization.
